# Integrating genetic data with biological insight: A practical guide to *cis*-Mendelian randomization

**DOI:** 10.1016/j.ajhg.2026.03.011

**Published:** 2026-04-10

**Authors:** Ville Karhunen, Benjamin Woolf, Pallav Bhatnagar, Dipender Gill, Stephen Burgess

**Affiliations:** 1 MRC Biostatistics Unit, School of Clinical Medicine, University of Cambridge, Cambridge, UK; 2 Sequoia Genetics, London, UK; 3 Eli Lilly and Company, Lilly Corporate Center, Indianapolis, IN, USA; 4 Cardiovascular Epidemiology Unit, Department of Public Health and Primary Care, University of Cambridge, Cambridge, UK

**Keywords:** Mendelian randomization, causal inference, drug target

## Abstract

*Cis*-Mendelian randomization is a computational approach that uses genetic variants from a biologically relevant gene region to assess the plausibility of a causal effect of a specific mechanism on an outcome of interest. As the use of *cis*-Mendelian randomization has recently increased due to the abundance of genetic association data for molecular quantitative traits, there is a need for informed guidance on how best to conduct such studies. Here, we review and discuss the key considerations for conducting robust *cis*-Mendelian randomization analyses. The main considerations include selection of the gene region of interest, the choice of the best traits to proxy the exposure of interest, variant selection and validation, and relevant sensitivity analyses. We highlight the importance of incorporating biological insight throughout the whole analysis process and that the analytical methods should be tailored to each gene region. Moreover, we point out some key differences from genome-wide Mendelian randomization—where variants are selected across the genome—and emphasize that *cis*-Mendelian randomization requires a distinct set of sensitivity analyses. We believe the advice we provide in this review will lead to a higher standard in planning, conducting, reviewing, and interpreting *cis*-Mendelian randomization studies.

## Graphical abstract

**Figure:**
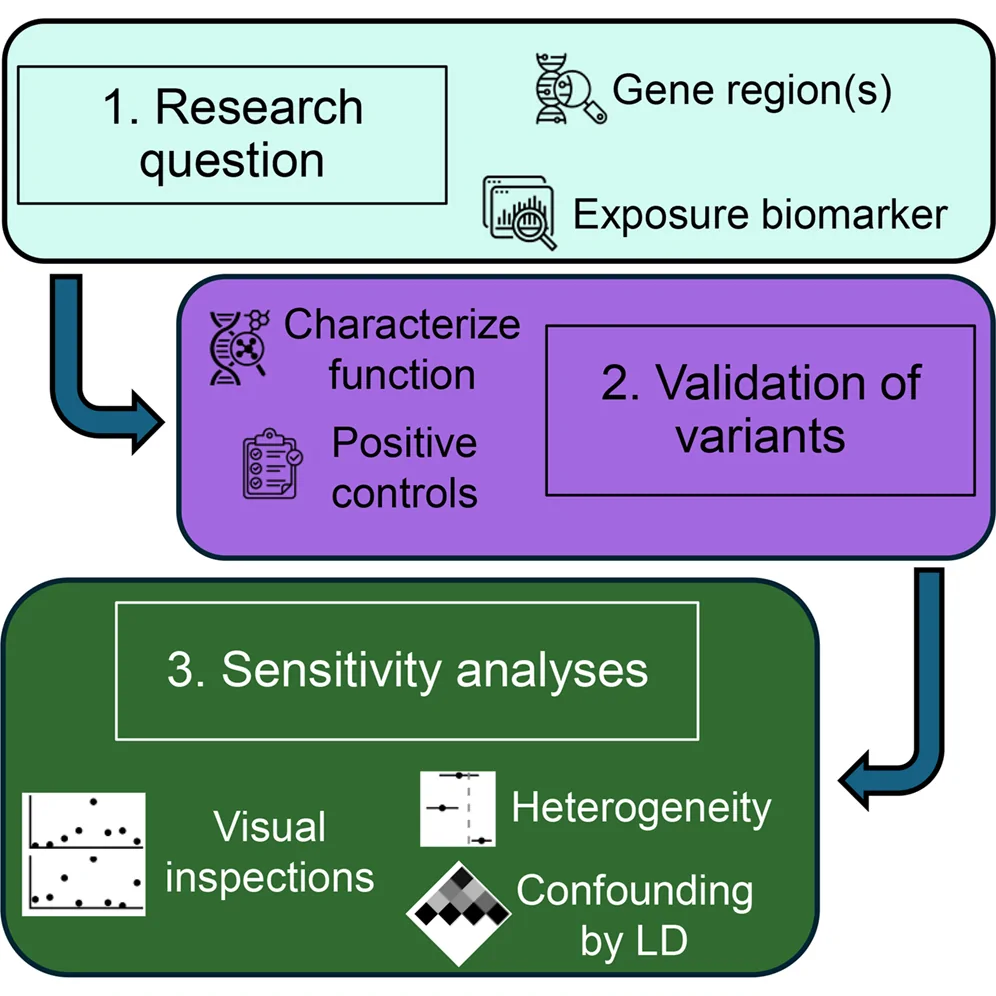

## Teaser

In recent years, the popularity of using *cis*-Mendelian randomization to assess causal mechanisms has increased due to the availability of genetic association data for molecular quantitative traits. In this review, Karhunen et al. discuss the key considerations and provide advice on the best practices for *cis*-Mendelian randomization studies.

## Introduction

Mendelian randomization (MR) is an approach that uses genetic variants—such as single-nucleotide polymorphisms (SNPs)—to assess the potential causal effect of an exposure on an outcome.^1^ MR can provide evidence for a causal effect if the genetic variants used fulfill the following instrumental variable assumptions: (1) relevance (the genetic variants used are robustly associated with the exposure), (2) independence (the association between the genetic variants and the outcome is not confounded), and (3) exclusion restriction (the genetic variants can influence the outcome only through the exposure).^2^

In *cis*-MR, the choice of variants is restricted to one or more biologically relevant gene regions, typically the gene region encoding the protein that is the exposure of interest. Such a gene region is known as the *cis*-gene region, hence *cis*-MR. While the original applications of MR were in the context of *cis*-MR,^3^^,^^4^ its popularity has recently increased due to the availability of genetic associations for large-scale molecular data and, in particular, circulating proteins.^5^^,^^6^ Moreover, as most pharmacological targets are proteins that are encoded by genes, the *cis*-MR approach is particularly useful for assessing drug target effects.^7^

*Cis*-MR can be considered in contrast to a more data-driven approach of selecting instruments across the whole genome, often using statistical criteria only—we refer to this as genome-wide MR. While these approaches are but two extremes of a continuum and there is nuance in the variant selection strategies, this crude categorization serves as a useful starting point for discussing *cis*-MR in detail.^8^

One of the key considerations in MR is pleiotropy. While the term pleiotropy is sometimes used in a positive sense in drug development to indicate the effect of a mechanism on multiple outcomes, we use the term to refer to effects of a genetic variant on exposure and outcome that act via different mechanisms and therefore violate the exclusion restriction assumption. A variant that is pleiotropic cannot reliably inform us about the impact of the exposure on the outcome. We highlight that the considerations about pleiotropic effects are different in *cis*-MR from those in genome-wide MR, which has implications for both the strategy to select genetic variants and the relevant sensitivity analyses in a *cis*-MR study.

Nowadays, MR is an established method in genetic epidemiology, and there are various guidelines for conducting MR studies.^8^^,^^9^ However, the focus of these instructional papers is mostly on genome-wide MR. Of those on *cis*-MR, Gill et al.,^7^ Schmidt et al.,^10^ and Holmes et al.^11^ consider *cis*-MR from a largely biological perspective, while Gkatzionis et al.^12^ and Burgess et al.^13^ provide a review of statistical methods with a focus on variant selection.

Here, we aim to provide a further perspective on how best to integrate biological and statistical insight in *cis*-MR. We consider issues in choosing the best trait to proxy the exposure of interest, selecting the variants, validating the variants, and performing sensitivity analysis. Our guidelines are based on general biological principles, and we use examples to illustrate the application of these principles to specific analytic questions.

## Choice of research question

### When to use *cis*-MR

The decision to conduct a *cis*-MR investigation should be driven by the research question. Generally speaking, if it is possible to conduct a biologically informed approach to variant selection, this would be preferred over an approach purely using statistical criteria.^14^ In reality, there is a spectrum of possible MR analyses ranging from those using variants in one gene region to those using hundreds of variants from across the genome. Therefore, there is a balance to be made between the specificity and generality of the question. Additionally, there is a trade-off between the statistical power (i.e., more variants would increase statistical power if they serve as valid instruments) and the validity of the instrumental variable assumptions (more variants means more opportunities for the instrumental variable assumptions to be violated).

For example, high levels of low-density lipoprotein (LDL) cholesterol have been identified as a causal risk factor for cardiovascular disease.^15^ 3-hydroxy-3-methylglutaryl-CoA reductase (HMGCR) inhibitors, commonly known as statins, are efficacious drugs in lowering LDL cholesterol levels.^16^ If a researcher is interested in investigating the effect of lowering LDL cholesterol levels via HMGCR inhibition on an outcome of interest, a *cis*-MR approach, restricting variant selection to the vicinity of *HMGCR*, is preferable. While LDL cholesterol levels are highly polygenic, the restriction of the variant selection to the *HMGCR* region can help ensure that the genetic associations are specific to HMGCR inhibition. However, if the research question considers the effects of lowering LDL cholesterol levels via any mechanism, genome-wide MR may be a more effective approach, as including more variants will typically increase statistical power. A further alternative would be to consider the effect of LDL-receptor inhibition using variants in genes related to receptor-mediated lipoprotein removal^17^; such an analysis could use variants in multiple gene regions that converge on this mechanism but not all genome-wide significant predictors of LDL cholesterol.

In general, a well-designed *cis*-MR is likely to be more reliable than a genome-wide MR, as *cis*-MR analyses are less prone to pleiotropy and more likely to fulfill the gene-environment equivalence assumption, which means that the effect on the outcome of changing the genotype would correspond to an equivalent effect of changing the exposure.^9^ While there is currently a lack of systematic evidence, there are clear examples of exposures for which the *cis*-MR analysis results differ from the genome-wide MR results, with the *cis*-MR results being more plausible from a biological perspective. For instance, MR investigations into the effect of C-reactive protein (CRP) levels on coronary artery disease (CAD) risk could give a wide range of answers, as some variants associated with CRP have positive associations with CAD risk, and others have negative associations.^18^ However, analyses based on variants in the region containing *CRP* have provided null findings.^19^ Similarly, a genome-wide MR suggested that circulating adiponectin levels are causally affecting type 2 diabetes risk.^20^ However, this finding was not supported by a subsequent *cis*-MR analysis using variants in the *ADIPOQ* (adiponectin, C1Q, and collagen domain containing) region only.^21^ As *ADIPOQ* codes for adiponectin, this analysis is less susceptible to pleiotropy and more likely to inform us about the causal impact of adiponectin.

### Choice of gene region

The core idea behind *cis*-MR is to use biological information to restrict the choice of the variants to the vicinity of a gene that plausibly affects the exposure in a specific way. This approach attempts to address conventional horizontal pleiotropy by design rather than in the analysis stage, as is usually done in genome-wide MR using pleiotropy-robust methods.^22^

The genomic region of interest should be relevant to the biological pathway under consideration, and therefore, the selection requires knowledge of the exposure mechanism of interest. The most common choice is the gene region that encodes the specific protein of interest. In some cases, multiple regions may be adequate (see examples below). Many of the considerations in this work are equally relevant to analyses based on variants in a single gene region (or a limited number of gene regions), even if the exposure is not a protein.

In the previous example of HMGCR inhibitors, a natural choice for selecting variants for *cis*-MR is *HMGCR*, as this gene encodes the corresponding protein. Alternatively, one could consider other relevant gene regions from the signaling pathway. An example of this is interleukin-6 (IL-6) signaling: IL-6 binds to its receptor (IL-6R), and genetic associations with relevant biomarkers (see next section) of IL-6 signaling are observed for variants within *IL6R*.^23^^,^^24^ Therefore, *cis*-MR investigations routinely use genetic variants within *IL6R* as proxies for IL-6 signaling.^25^^,^^26^ More recently, broadly similar conclusions have been reached using variants within the *IL6* region.^27^

While *cis*-MR is usually conducted using variants from a single gene region, for some exposures, multiple regions can be considered biologically relevant. For example, calcium channel blockers are a class of drugs that treat various cardiovascular conditions. They inhibit multiple related proteins that are encoded by different gene regions. If the aim of an MR study is to assess the effect of calcium channel blockers, then variants can be considered from any of these gene regions.^28^

In other cases, the exposure of interest may not be a protein, but the analysis is still based on one or more biologically relevant gene regions. For example, MR analyses with alcohol consumption as the exposure may use variants in regions containing aldehyde dehydrogenase 2 family member (*ALDH2*) and/or alcohol dehydrogenase 1B (class I), beta polypeptide (*ADH1B*), which encode key proteins in the metabolism of alcohol and its byproducts.^29^ Similarly, MR analyses with vitamin D as the exposure have used variants in four gene regions that encode key players in the synthesis and metabolism of 25-hydroxyvitamin D.^30^

The advantage of using a single gene region is its simplicity. Multiple genes, such as ligand and its receptor, can be used in some scenarios^31^ but only when the genetic associations with relevant biomarkers in both of these loci have been ascertained to be proxying the same mechanism of interest. While the use of multiple genes may improve statistical power, it opens up more possibilities for instrument heterogeneity or confounding by linkage disequilibrium (LD; see sections below).

### Choosing the exposure biomarker

After identifying the gene region(s) of interest, a crucial aspect of *cis*-MR is the selection of an exposure biomarker, that is, a trait that effectively proxies the true exposure of interest. In practice, the steps of choosing the gene region and the exposure biomarker can partially overlap, as the lack of strong genetic associations with an exposure biomarker within one gene region may indicate the absence of adequate variants within that region to serve as instruments. There are usually various options in choosing the exposure biomarker. For example, to proxy HMGCR inhibition, one could select variants that strongly associate with *HMGCR* expression (expression quantitative trait loci [eQTLs]), circulating HMGCR levels (protein QTL [pQTL]), or a known downstream consequence (endophenotype) of HMGCR inhibition, such as LDL cholesterol levels. In some cases, the same variants may be the strongest predictors of gene expression, protein levels, and downstream endophenotypes. However, in other cases, variants associated with these traits may not overlap.

**Figure 1:**
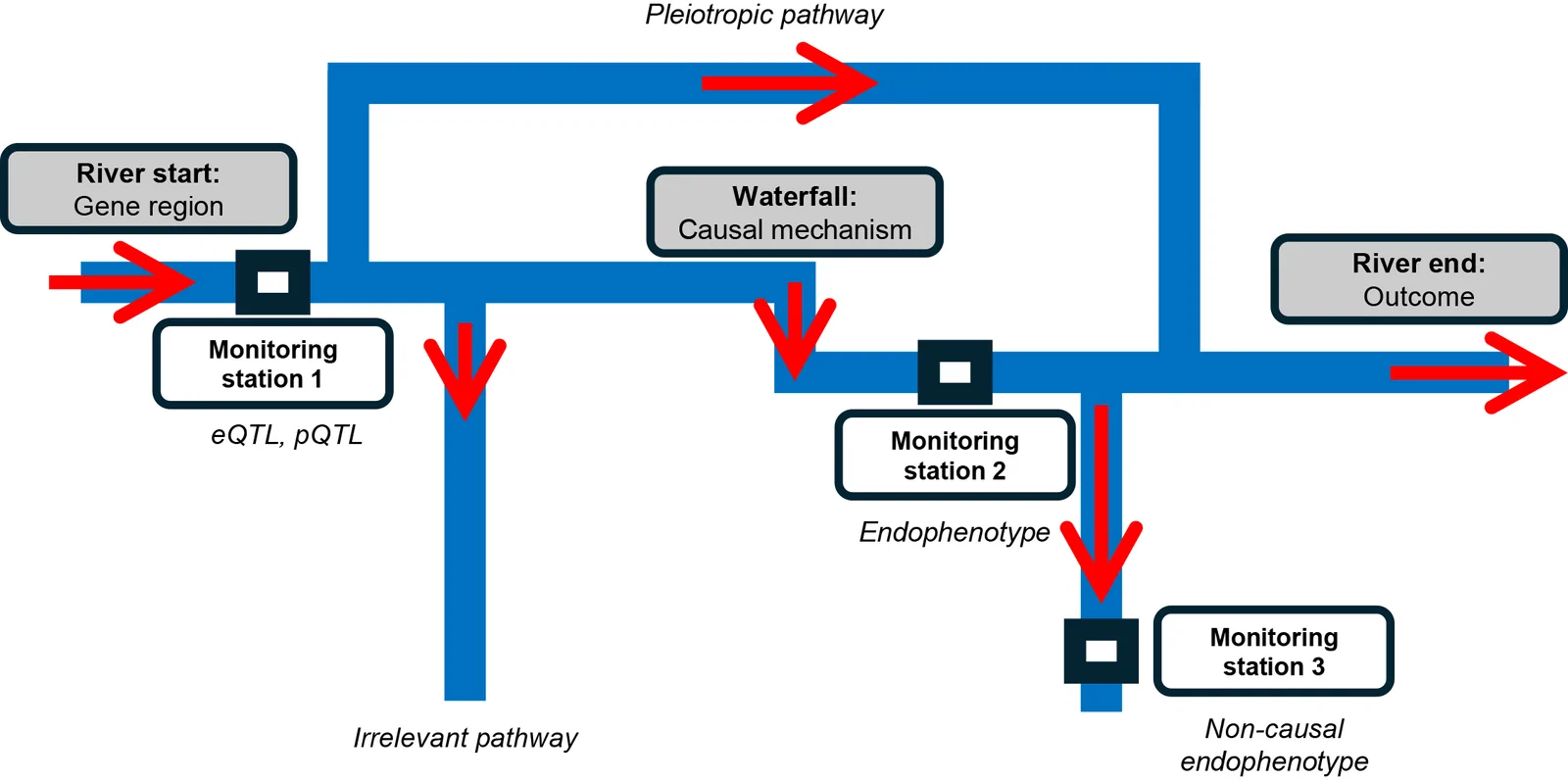
Different options for choosing the exposure biomarker to proxy the exposure of interest in *cis*-Mendelian randomization, with an analogy to a river with a waterfall The aim is to measure the quality of water that passes through the waterfall (this is analogous to the causal mechanism of interest). Water quality can be measured at different monitoring stations (analogous to different exposure biomarkers). Water sampled upstream of the waterfall at monitoring station 1 (analogous to an exposure biomarker proximal to the gene, such as gene expression or protein levels) may not pass through the waterfall. Instead, it may not reach the waterfall (irrelevance) or may bypass it (pleiotropy). In contrast, water sampled downstream of the causal mechanism at monitoring station 2 (analogous to a downstream endophenotype as the exposure biomarker) must have passed through the waterfall. Moreover, water sampled downstream at monitoring station 3 must have passed through the waterfall even though it does not reach the river end (analogous to a downstream biomarker or endophenotype not being on the causal pathway to the outcome).

When there are different possibilities for the exposure biomarker, we argue that a trait that is *downstream of* the exposure mechanism of interest—or conversely, a trait close to the outcome of interest—is most likely to satisfy the MR assumptions (Figure 1). While this may sound counterintuitive at first, this strategy minimizes the potential for biological pleiotropic effects that violate the exclusion restriction assumption and irrelevant variants that do not proxy the mechanism under investigation.^32^^,^^33^

**Figure 2:**
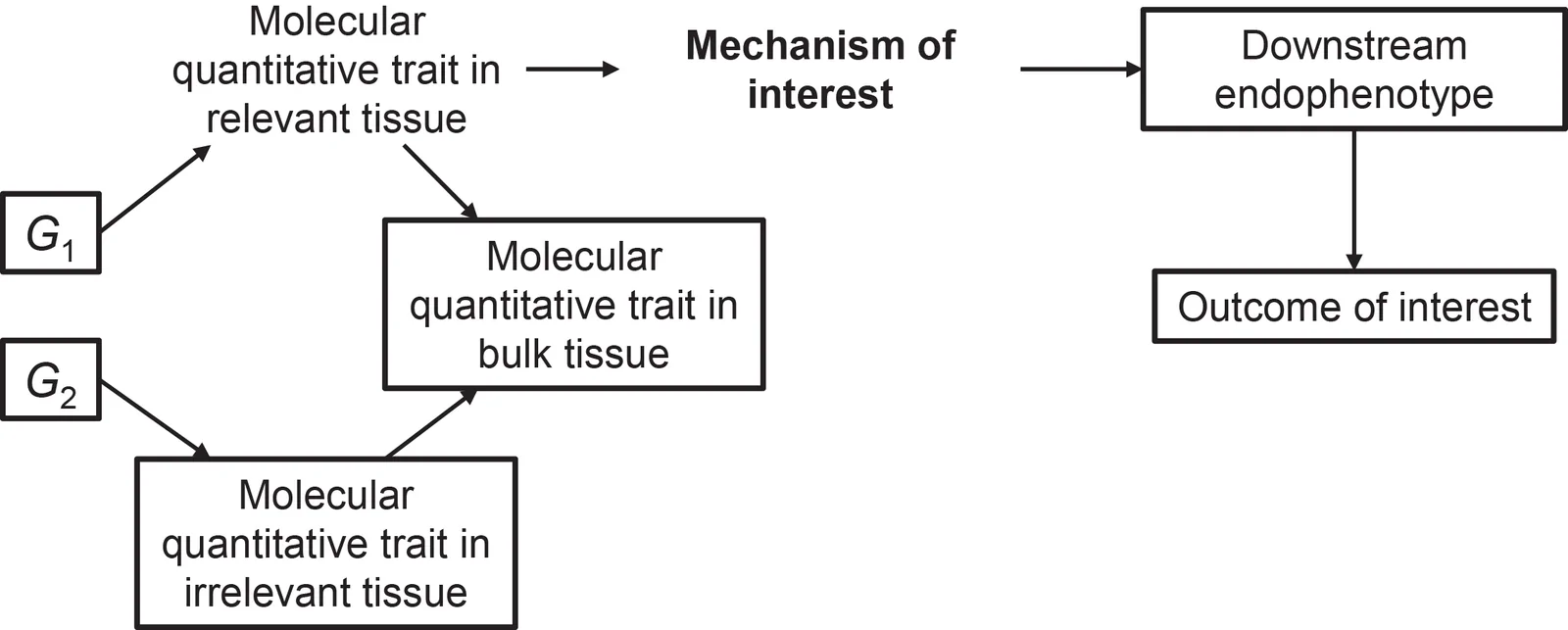
An illustration of choosing an adequate exposure biomarker to proxy the putative causal mechanism of interest in *cis*-Mendelian randomization Boxes indicate measured variables; *G* refers to genetic variants. If the exposure biomarker is a molecular quantitative trait, such as gene expression or protein levels, and it is measured in an irrelevant tissue or based on bulk tissue, a genetic variant *G*_*2*_ that is associated with these quantities may not affect the mechanism of interest. However, if a downstream endophenotype is selected as the exposure biomarker, any variant *G*_*1*_ that is associated with this biomarker will affect the putative causal mechanism (see also Note S2).

For example, both gene expression and protein levels are known to be tissue specific, and if either of them is used as the exposure biomarker for choosing the variant(s) but the genetic associations are not measured in a relevant tissue, then the variant(s) may not affect the true exposure mechanism of interest. In contrast, if the exposure biomarker is selected as a downstream endophenotype of the exposure mechanism, then any *cis*-variants that associate with this exposure biomarker will also associate with the true exposure of interest and therefore truly mimic the exposure (Figure 2). In the HMGCR example, the strategy outlined here would favor using LDL cholesterol levels as the exposure biomarker.

In Figure 1, we make an analogy of choosing the exposure biomarker for *cis*-MR to a river with a waterfall: the river represents the flow of information from the gene (the starting point of the river) to the outcome (end of the river), with a waterfall in the middle representing the biological mechanism of interest. In the analogy, if the interest is in measuring the quality of the water running through the waterfall, a measurement sample taken from a location shortly downstream will have run through the waterfall. However, a measurement taken upstream of the waterfall may not always reach the waterfall but could be lost to other streams. Similarly, when we aim to proxy the causal mechanism of interest, an exposure biomarker that is shortly downstream of the mechanism of interest is likely to capture the effect of the causal mechanism of interest, while a measure upstream of the mechanism (such as eQTLs or pQTLs) may be lost to different pathways that are irrelevant to the mechanism of interest. In the analogy, we assume that we are interested in one particular route from the start (gene) to the end (outcome); in the case of drug-target MR, this would be the druggable mechanism. There may be pleiotropic effects directly from the gene to the outcome that do not relate to the mechanism of interest. However, such effects can be mitigated by the adequate selection of the exposure biomarker specific to the mechanism of interest.

#### Exposure biomarker can differ from the causal agent

We emphasize that in the context of drug-target MR, the exposure biomarker itself does not need to be the causal agent as long as the direction of the effect of drug target perturbation on the exposure biomarker is known (Note S1).^34^^,^^35^ The choice of the causal question is defined by the selected gene region, and the exposure biomarker simply indicates the effect of perturbing the exposure mechanism of interest.

In the IL-6 signaling inhibition example, CRP levels are a known biomarker for IL-6 signaling inhibition,^36^ and they have been used as the exposure biomarker in MR studies that show evidence for a protective effect of this mechanism on severe COVID-19 risk,^37^ consistent with clinical trials.^38^ However, CRP itself is not believed to be on the causal pathway from IL-6 inhibition to severe COVID-19.^39^

As another example, in a recent work by Rahu et al.,^33^ the authors assess the effects of histidine ammonia-lyase (HAL) on skin cancer risk. HAL is an enzyme converting histidine to *trans*-urocanate that acts akin to a natural UV protector on skin and has a role in regulating vitamin D levels.^40^^,^^41^ Despite no evidence of vitamin D levels being causal to skin cancer risk,^42^ variants within *HAL* that associate with vitamin D levels (that is, using vitamin D levels as the exposure biomarker) could be used to assess the effect of HAL on skin cancer risk.

A non-genetic analogy would be an examination of the effect of temperature on heat stroke risk. We know that temperature (the true exposure in our analogy) causes expansion of mercury in a thermometer (the exposure biomarker), and therefore, we can use mercury volume as a surrogate measure for temperature variation. However, we would not infer that the mercury volume within the thermometer is the causal agent increasing heat stroke risk (our outcome of interest).

#### Caution when using QTL data as the exposure biomarker

Suitable downstream endophenotypes may not always be available or known; in that case, one can consider eQTL or pQTL data as the exposure biomarker. However, additional care must be taken in these cases.^32^ Some possible problems when using molecular QTL data for variant selection include cell-type-specific, dynamic, or context-specific effects,^43^^,^^44^^,^^45^^,^^46^ protein abundance measurement artifacts due to protein-truncating missense variants,^47^^,^^48^ and pleiotropic effects due to *cis*-co-regulation^48^^,^^49^ or *trans-*acting effects.^50^ There is also a known discrepancy between the types of variants that are hits for gene expression and those for complex traits in genome-wide association studies (GWASs), suggested to be due to different types of selective constraints.^51^ Likewise, the variants that most strongly associate with tissue-specific expression or circulating protein levels may not be variants that impact protein function. All these phenomena may lead to variants being either pleiotropic or irrelevant (Figure 1), leading to false positive or false negative associations.

For example, large-scale molecular QTL data are often available only for easily accessible tissues, such as blood,^52^ or as a bulk measurement across different cell types.^53^ While the genetic associations for such tissues willtypically be stronger than those with a downstream endophenotype, they will be less specific. If the exposure biomarker is measured in an irrelevant tissue or in an aggregate measure of multiple tissues (or cell types), then our variants may not be associated with the outcome even if there is a true causal effect. In contrast, if the exposure biomarker is selected downstream of the correct but potentially unavailable tissue, then the test for the null hypothesis of no causal effect is valid (Figures 2 and S1; Note S1).^35^

### Choice of genetic variants

The best candidates for variants in *cis*-MR are functional variants with a well-known biology. For example, the missense variant rs34536443 is known to affect the enzymatic activity of tyrosine kinase 2 (*TYK2*),^54^^,^^55^^,^^56^ with consistent protective effects across autoimmune diseases.^56^ Therefore, this variant has a strong justification to be used within the MR framework to proxy TYK2 inhibition.^57^^,^^58^ Missense variants, which change the amino acid and consequently may lead to functional changes in the protein, have been used in *cis*-MR to proxy different types of exposures, such as iron homeostasis,^59^ inflammation,^26^^,^^60^^,^^61^^,^^62^ and malaria.^63^ Another advantage of using missense variants in *cis*-MR is that they are less likely to have tissue- or cell-type-specific effects, which is useful when the exposure of interest is a target anticipated to have systemic effects. However, not all missense variants necessarily affect the exposure mechanism of interest, and they can also be liable to measurement artifacts in aptamer-binding-based methods, where the binding affinity of the protein may be changed without protein abundance.^48^ Hence, rigorous justification for a valid missense variant in *cis*-MR is always warranted.

In the absence of such well-characterized functional variants, one would need to rely on more statistically driven approaches. However, we emphasize the importance of adapting instrument selection criteria for each specific scenario, with a focus on biological justification for the variant(s) based on all available evidence rather than overreliance on a single statistical criterion, such as a specific *p* value threshold. In some cases, there may not be a suitable genetic variant to perform *cis*-MR, even if a relevant gene region is identified.^64^

Statistical methods for choosing genetic variants from a single gene region have been previously described in detail.^12^ Briefly, variant selection methods based on the genetic associations with the chosen exposure biomarker include clumping of correlated variants, fine-mapping methods, and dimension-reduction-based approaches. We here discuss design choices that have not been highlighted elsewhere.

First, in *cis*-MR variants can arguably be selected at a gene-region-wide significance level (e.g., *p* < 5 × 10^−4^ if there are 100 independent candidate variants) rather than a genome-wide significance level. However, using a lenient *p* value threshold is appropriate only when there is a strong biological justification and further support for the relevance of the variant. We do not advise routinely using such a lenient threshold, especially without further validation. Furthermore, the lenient threshold for variant selection needs to be balanced with a potential increase in weak instrument bias^65^ or winner’s curse bias.^66^

Second, using multiple variants solely to enable the use of robust methods for sensitivity analysis is not advised. It is generally better to use a single reliable variant that clearly mimics the relevant causal mechanism rather than several genetic variants for which the relevance is unclear. Indeed, *cis*-MR is often conducted using a single variant,^26^^,^^57^^,^^61^^,^^62^^,^^63^^,^^67^ and particularly if multiple variants are selected using statistical criteria only, it may become difficult to have certainty of the mechanism of each variant. The fundamental balance is between signal and noise: more signal is better, but there should be a high bar for increasing the signal when this risks adding more noise. Additionally, the statistical associations with the chosen exposure biomarker do not necessarily predict instrument strength. This may happen when, for example, the variants associate with protein levels rather than protein function.

For example, in a study assessing the renoprotective effects of fibroblast growth factor 21 (FGF21), Giontella et al.^68^ identified a missense variant rs739320 within *FGF21*, which associated with estimated liver fat percentage (the exposure biomarker) at a region-wide significance level, and further showed associations with other relevant biomarkers as well as positive control traits (see below for further considerations on variant validation). A variant with strong biological justification for proxying the mechanism of interest, as judged by the functional role of the mutation (such as being a missense variant) or its associations with proposed downstream consequences of intervention in the mechanism, may be preferred over a variant that has stronger statistical associations but does not have biological support.

### Window size

It is typical to select *cis*-acting variants not only from within the gene of interest but from a region around the target gene region known as a window. The reason for this is that measured variants just outside the gene may serve as valid proxies for the exposure of interest. The ideal size of the *cis*-region to be investigated heavily depends on the particular genomic region (see Box 1 for further discussion and examples^69^^,^^70^). In a gene-dense area, variants outside of the coding region for the protein of interest might impact nearby genes. In this setting, it may be better to be strict with the definition of a *cis*-signal, as there is a higher risk of pleiotropic signals from nearby genes. In contrast, in genomic regions with few genes, one may be more lenient with the window. Assessing regional association plots for the relevant biomarkers and phenotypes helps assess the location and specificity of the genetic signal of interest. Additionally, statistical approaches such as fine-mapping—where the aim is to detect independent variants that are associated with the phenotype(s) of interest, accounting for LD patterns—may offer insight into the genetic associations within the region.^71^^,^^72^^,^^73^

Box 1 **Determining the ideal window size**

In *cis*-MR, the genomic window used to select the variants varies from case to case. Aspects to keep in mind when choosing an appropriate window include the number and location of genetic signals for relevant biomarkers and phenotypes within the locus, linkage disequilibrium patterns within the region, and nearby genes and their function in relation to the exposure mechanism and the outcome of interest. As these characteristics vary from one gene region to another, it is important to customize the definition of the *cis*-gene region for each scenario. The locations of enhancer or promoter regions can also be considered. In some instances, such as when the gene of interest lies in a gene-rich region with other nearby genes that may plausibly have an effect on the outcome, the best choice may be to restrict the analysis strictly within the gene. In other cases, a wider window of up to ±250 kb may be appropriate.

It can be useful to initially visualize a large genomic window, say ±1 Mb from the gene of interest, and then infer on the optimal window size based on the aspects above. Furthermore, fine-mapping—a statistical approach to detect independent signals for the variable of interest, adjusting for linkage disequilibrium patterns—can be used to disentangle the genetic signals for different biomarkers and phenotypes within the locus.

If there is a concern that nearby genes might potentially confound the association between the causal mechanism of interest and the outcome, then one should opt for a stringent definition of a *cis*-region. For example, Kang et al.^69^ assessed the effects of tumor necrosis factor inhibition on Parkinson disease, where they identified their instruments from the vicinity of *TNFRSF1A* (TNF receptor superfamily member 1A) with a ±10 kb window. Their reasoning for the narrow window was that the genomic region contains other immune-related genes that may be associated with the outcome risk independently of the exposure mechanism of interest. In cases where there is less ambiguity, a window such as ±100 kb is common.^70^

## Validation of the genetic variants

As discussed above, the ideal approach to validate a genetic variant is to characterize its biological function and use this to justify the MR assumptions. Furthermore, when effects of intervening on the exposure of interest are known (e.g., from clinical studies/trials), then it is advisable to check that the variants associate with any positive control outcomes, which can be biomarkers for the mechanism of interest,^74^ or outcomes that intervention on the proxied mechanism is expected to affect. For example, in a study using variants in phosphodiesterase 5A (*PDE5A*) to proxy the mechanism of the drug sildenafil (a PDE5A inhibitor), Woolf et al. validated their instruments using pulmonary arterial hypertension and erectile dysfunction (two conditions that sildenafil is licensed to treat) as positive controls.^75^

Negative control outcomes are outcomes that intervention on the proxied mechanism is not expected to affect. Similarly, negative control populations are those in which intervention on the proxied mechanism is not expected to affect. These can demonstrate that the associations of the genetic variants are specific to the drug target of interest, reducing the possibility of pleiotropic effects. As an example, in the PDE5A work introduced above, the authors used female participants as a negative control population when considering the effect of PDE5A inhibition on reproductive outcomes.^75^

## Statistical analyses

In general, the primary statistical analysis methods in *cis*-MR differ little from genome-wide MR. If the analysis is based on a single genetic variant, analysts should use the ratio method, with either first- or second-order delta method approximation of the standard error.^76^ If investigators are interested in the magnitude of the MR estimate, then using the second-order approximation of the standard error is more appropriate, as it accounts for uncertainty in the genetic association with the exposure. However, if the investigator is solely interested in whether there is evidence for a causal effect, then the first-order approximation is reasonable. A statistically equivalent approach with the first-order approximation is to simply report the genetic association with the outcome. The benefits of framing this as a *cis*-MR investigation are to underline the importance of robustly establishing that the variant proxies the exposure mechanism of interest and that the MR estimate can be expressed in units per genetically predicted change in the exposure.

With summarized data on multiple variants, the inverse-variance weighted method is recommended as the primary analysis method in both *cis*-MR and genome-wide MR, as it most efficiently combines the evidence from multiple genetic variants, provided that the variants fulfill the instrumental variable assumptions.^77^ If variants are partially correlated, then a variation on this method that accounts for variant correlation (using an adequate genotype correlation reference) should be used.^78^

A characteristic of *cis*-MR is that there are usually fewer variants available than in genome-wide MR. Hence, while with many genetic variants, it is recommended to use a random-effect model for the inverse-variance weighted method, with few variants, estimates of between-variant heterogeneity may be unstable. Further, for this and other reasons discussed in the next section, many standard sensitivity analyses for genome-wide MR have little utility for *cis*-MR. In genome-wide MR, relying on statistical approaches to assess the validity of genetic variants as instruments is never ideal, but these approaches can provide insights; in *cis*-MR, several of these approaches cannot even be attempted.

### Pleiotropy-robust methods

Numerous MR methods that are robust to some pleiotropic effects have been developed and routinely applied in the context of genome-wide MR.^79^ The most commonly used pleiotropy-robust methods can be crudely categorized into methods based on the consensus of evidence, such as median-based methods, mode-based methods, and MR-PRESSO (MR pleiotropy residual sum and outlier),^80^^,^^81^^,^^82^ or methods based on modeling the distribution of pleiotropic effects, such as MR-Egger, MR-RAPS (MR robust adjusted profile score), or MR-cML (MR constrained maximum likelihood).^83^^,^^84^^,^^85^ All these methods rely on evidence from multiple genetic variants that are candidate instruments.

In genome-wide MR using uncorrelated variants in distinct gene regions, pleiotropic effects are plausibly independent of each other if they occur via different pathways. In such a case, the bias in each variant’s individual MR estimate should be specific to that variant, known as “idiosyncratic pleiotropy.” Pleiotropy in genome-wide MR can therefore be thought of as a source of heterogeneity in variant-specific MR estimates.^86^ Methods based on consensus of evidence typically assume that any pleiotropic biases from each variant are either rare or independent of each other, with an average effect of zero.

Since variants in *cis*-MR studies are often located within the same gene region, pleiotropic effects are unlikely to be independent. If there is pleiotropy in the mechanism of a gene, then all variants in that gene region are likely to suffer from similar pleiotropic errors and hence would be systematically biased even if the variants are uncorrelated with each other. As such, sensitivity analyses that rely on consensus of evidence are unlikely to be useful in the context of *cis*-MR since they cannot detect systematic violations of the instrumental variable assumptions. Additionally, several of these methods assume that the genetic variants are uncorrelated, and so their estimates would typically be overly precise if implemented with partially correlated variants.

As an example, we provide a *cis*-MR analysis of apolipoprotein C-I (APOC1) signaling on Alzheimer disease risk, using total cholesterol levels as the exposure biomarker (details are provided in Note S2). This analysis provides strong evidence for APOC1 signaling increasing Alzheimer disease risk (inverse-variance weighted odds ratio estimate per 1-standard-deviation increase in genetically proxied cholesterol levels 3.17, 95% confidence interval: 2.05–4.91), with concordant effect size estimates from weighted median and weighted mode methods (Table S1). However, the instruments used in this example are all in LD with rs429358 in the neighboring gene, *APOE*, a well-known variant that affects Alzheimer disease risk (Table S2). This leads to systematic errors that are not captured by the pleiotropy-robust methods.

A separate issue arises when using modeling-based robust analysis methods in *cis*-MR. The model parameters are more accurately estimated when many data points are available. In the case of only a few available genetic variants (i.e., data points), the standard errors for the parameters will be very wide, and any inferences based on these methods will be imprecise. Statistical tests, such as the MR-Egger intercept test for pleiotropy, will likely be underpowered to detect any departures from the null.

When implemented and interpreted correctly, use of pleiotropy-robust methods in *cis*-MR is not harmful, but it is unlikely to be particularly informative. Robust methods may be more useful in analyses based on variants from multiple gene regions. A leave-one-out approach, where all variants from each gene region are omitted in turn, may also be worthwhile.

There are also data-driven pleiotropy-robust methods that have been developed specifically for *cis*-MR settings. The MR-link-2 method estimates the causal effect of the exposure on the outcome using all variants in the region while treating pleiotropic variance as a nuisance parameter^87^ and is conceptually related to combining MR and proportional colocalization (see section below) into a single workflow. The *cis*-MR-cML method applies variable selection to detect variants associated with either the exposure or the outcome and uses constrained maximum likelihood to estimate the causal effect based on the selected variants’ mutually conditioned associations.^88^

### Heterogeneity

Heterogeneity refers to the variability in variant-specific MR estimates that exceeds the variability expected due to chance only. If multiple variants are available, statistical measures of heterogeneity in the variant-specific estimates, such as *I*^2^, can be worthwhile as an indicator that some variants may be invalid instruments, that is, violating the instrumental variable assumptions. Heterogeneity indicating variant invalidity is demonstrated in the work by Tambets et al.,^48^ where the authors conducted a systematic benchmarking of eQTL-pQTL associations within their coding genes via *cis*-MR. In their analyses, the observed heterogeneity in the variant-specific MR estimates was likely to be caused by co-regulation of nearby genes and therefore pleiotropic variants, additionally demonstrating the pitfalls of using eQTL variants in *cis*-MR.

However, we caution that heterogeneity does not necessarily imply invalidity. It may instead mean that the exposure biomarker does not fully reflect the change in the causal mechanism attributable to a variant (and therefore is not a fully accurate exposure biomarker). For example, if the exposure biomarker is levels of a protein, then it may not capture the impact of a variant that changes protein function; in line with this, the aforementioned variant rs34536443 in *TYK2* associates only weakly with plasma TYK2 levels (*p* = 0.019),^5^ suggesting that protein levels are not the appropriate exposure biomarker in this case. Conversely, failure to detect heterogeneity does not imply validity, either because power to detect heterogeneity is low or because variants could all be invalid in a similar way and hence do not display heterogeneity.

If multiple gene regions have been assessed, heterogeneity between the region-specific estimates can also provide insights into the causal mechanism pathway. For example, Yang et al. studied the effects of different LDL-cholesterol-lowering pathways on gallstone disease risk.^89^ Separate estimates were obtained for seven different gene regions, with circulating LDL cholesterol levels as the exposure biomarker. The results for some gene regions (such as *HMGCR*) indicated that lowering LDL cholesterol levels reduces the risk of gallstones, while the results for other gene regions (such as the region containing ATP-binding cassette subfamily G members 5 and 8 [*ABCG5* and *ABCG8*]) suggested that lowering LDL cholesterol levels increases the risk of gallstones. However, some gene regions lower cholesterol systemically, whereas others affect cholesterol transport. Gallstones are primarily composed of undissolved cholesterol, and therefore, the causal risk factor in this case is not circulating LDL cholesterol in the bloodstream but rather cholesterol in the gallbladder. Variants that affect cholesterol transport and associate with lower LDL cholesterol in the bloodstream are increasing cholesterol levels in the gallbladder. This aligns with trial evidence on ATP citrate lyase inhibitors, which lower LDL cholesterol in the bloodstream but elevate gallstone risk.^90^ For all gene regions, circulating LDL cholesterol is a valid exposure biomarker for variant selection, but the effect on the outcome of interest differs depending on the mechanism by which circulating LDL cholesterol is lowered.

### Confounding by LD and colocalization

Two variants are said to be in LD if they are correlated in their distributions within a population. Confounding by LD occurs when two separate genetic variants, one causal to the exposure and the other to the outcome, are in LD with each other, which may induce a false positive MR estimate.

Colocalization methods can assess the possibility of confounding by LD in *cis*-MR.^91^ In colocalization, the aim is to investigate whether genetic associations within a specific gene region for two traits are driven by the same causal variants or by distinct causal variants. The two main categories of colocalization methods are enumeration colocalization and proportional colocalization.

Enumeration colocalization explores different configurations of causal variants, typically within a Bayesian modeling framework. The most common method for colocalization is *coloc*, a Bayesian enumeration colocalization method that assumes that there is a maximum of one causal variant within a gene region for each trait.^92^ Under this assumption and given prior probabilities for a variant being associated with each trait, the method evaluates the posterior probabilities (PPs) for the hypotheses: H0, there is no causal variant for either trait; H1, there is a causal variant for trait 1 only; H2, there is a causal variant for trait 2 only; H3, there are distinct causal variants for traits 1 and 2 (i.e., non-colocalization); and H4: there is a common causal variant for both traits (i.e., colocalization).

When *coloc* is used as a sensitivity analysis for *cis*-MR, the exposure and outcome are usually referred to as traits 1 and 2, respectively. As a positive MR result implies that there is a causal variant for both traits, the main interest in *coloc* is whether *PP*.*H*4 > *PP*.*H*3, that is, whether evidence supporting a shared causal variant is stronger than evidence supporting distinct causal variants. A known limitation in *coloc* is that if the genetic associations with the outcome are weak, then the largest PP will be assigned to H1. Therefore, *PP*.*H*3 + *PP*.*H*4 will be small, and the method is underpowered to detect evidence for or against confounding by LD. A case where *PP*.*H*3 + *PP*.*H*4 > 0.5 represents the situation where a causal variant for both traits (either shared or distinct) is the more likely scenario than no causal variant for one of the traits and therefore suggests sufficient power for the *coloc* method to infer the plausibility of confounding by LD. If *PP*.*H*3+*PP*.*H*4 < 0.5, then investigators may consider *PP*.*H*4/(*PP*.*H*3+*PP*.*H*4), which represents the PP for colocalization conditional on the existence of causal variants for both traits. However, the relative evidence for H4 should be considered together with the absolute magnitude of the PPs—if *PP*.*H*3 + *PP*.*H*4 is close to zero, there is little evidence either way.

Another limitation of the *coloc* method is the assumption of at most one causal variant per trait within the gene region, which may not always be biologically realistic. Methods related to multi-trait fine-mapping, such as *coloc-SuSiE*,^93^ can be used to perform colocalization, allowing for multiple causal variants. In fact, the *coloc* method can be considered as simultaneous fine-mapping of two traits under the assumption of at most one causal variant per trait. Multi-trait fine-mapping methods using summary data such as *coloc-SuSiE* require accurate information about the LD structure to perform fine-mapping reliably. In practice, accurate LD information may be very hard to obtain, particularly for non-European ancestry populations, and therefore, these methods can be prone to inaccurate findings.^94^^,^^95^

Proportional colocalization evaluates whether the genetic associations for two traits within a gene region are similar up to a proportionality constant.^96^^,^^97^ A similar idea is implemented in the HEIDI (heterogeneity in dependent instruments) method, which tests the homogeneity of the variant-specific MR estimates within a gene region^98^ and, furthermore, can be used to exclude instruments that exhibit heterogeneity.^99^

The main limitation of proportional colocalization methods is that the null hypothesis is colocalization, and therefore, a lack of evidence to reject the null may indicate either true colocalization or a lack of power to detect evidence against it. To address this, Patel et al. proposed a refined proportional colocalization method with an additional test to determine whether the proportionality constant equals zero.^100^ A recent method, colocPropTest,^101^ also adopts the proportional colocalization framework and attempts to account for potentially varying sample sizes across variants and to identify false positives where the *coloc* method suggests confounding by LD (i.e., high *PP*.*H*3).

We explicitly highlight that colocalization as a sensitivity analysis in MR is not conducted to investigate horizontal pleiotropy, since, in the presence of colocalization, horizontal pleiotropy and vertical pleiotropy (i.e., an effect fully mediated by the exposure) cannot be distinguished from each other using statistical criteria only. Instead, colocalization is conducted to investigate potential confounding by LD. Further, colocalization methods are not helpful if the genetic associations with the chosen exposure biomarker do not adequately capture the true causal mechanism of interest.

### Conditional analysis

An alternative approach to investigate confounding by LD is a conditional analysis, where we consider the association of a genetic variant with the outcome conditional on another variant. For example, a functional variant in gastric inhibitory polypeptide receptor (*GIPR*) is associated with CAD risk, potentially providing insight into the effects of GIPR signaling on CAD risk. However, *GIPR* neighbors *APOE*, and APOE influences circulating levels of pro-atherogenic lipids. When considering the association of the *GIPR* functional variant conditional on a variant in the *APOE*, the association with CAD risk attenuates to the null.^102^ This provides persuasive evidence that the genetic association is biased due to confounding by LD. Conditional analyses can be performed based either on individual-level data by adjusting the regression model or on summarized data using the COJO (conditional and joint association analysis) tool.^103^ Similarly to other fine-mapping-related methods using summarized data, COJO is also highly sensitive to the chosen reference LD information.

### Visual inspections

**Figure 3:**
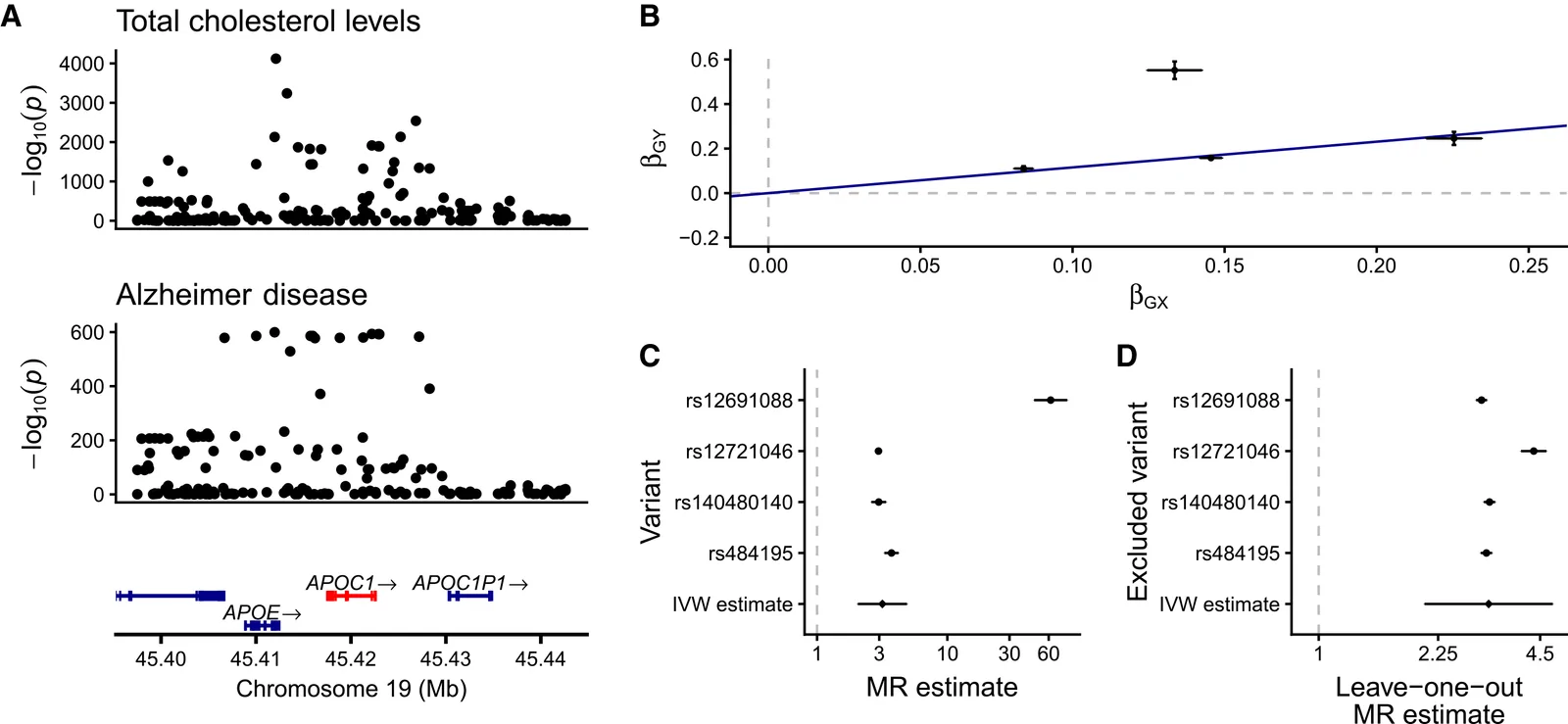
Examples of visual inspections for *cis*-Mendelian randomization The example is showing a *cis*-Mendelian randomization of apolipoprotein C-I (APOC1) signaling on Alzheimer disease risk (Note S2). We use total cholesterol levels as the exposure biomarker for APOC1 signaling. (A) Regional plots of the genetic associations for the traits. (B) Scatterplot of variant-outcome associations (β_GY_, log-odds ratio) against variant-exposure associations (β_GX_). The lines across the dots depict 95% confidence intervals, and the solid blue line represents the inverse-variance weighted (IVW) estimate. (C) Forest plot of the variant-specific Mendelian randomization (MR) estimates and their 95% confidence intervals. (D) Leave-one-variant-out MR estimates and their 95% confidence intervals. The figures suggest that variant rs12691088 is a source of heterogeneity, as the MR estimate for this variant is not consistent with the estimates for other variants. Excluding variant rs12720146 would lead to a notable increase in the overall IVW MR estimate. The reasons for heterogeneity should be carefully investigated. Note that this example is for illustration purposes only—there are systematic errors in the MR estimates as all four variants are in linkage disequilibrium with rs429358, a known risk variant for Alzheimer disease (Table S2).

We emphasize the importance of different visual inspections when conducting *cis*-MR analyses (Figure 3). Locus plots (regional association plots; Figure 3A), which depict the genetic associations with a trait for all variants within the genomic locus, can be valuable in assessing whether the genetic associations for the exposure and outcome align with each other (i.e., colocalization) or if there is evidence for confounding by LD.

When multiple variants have been used in MR, scatterplots of variant-outcome against variant-exposure associations (Figure 3B) and forest plots of the variant-specific MR estimates (Figure 3C) can be used to visually explore the consistency and heterogeneity of the estimates. Ideally, the variant-specific estimates should be directionally consistent with no excess deviations from the average effect. Forest plots from leave-one-variant-out analysis (Figure 3D) can help in assessing if the MR results are overly sensitive to the inclusion of a specific variant. In both cases, one should carefully consider the source of heterogeneity—we refer to the discussion in heterogeneity.

### Other *cis*-MR methods

Some extensions that have been developed for MR to address more complex questions have also been adapted to the *cis*-MR setting. If there is a plausible pleiotropic phenotype and/or confounding by LD, then *cis*-multivariable MR (*cis*-MVMR) or two-step *cis*-MR could be used to adjust for pleiotropic effects.^104^^,^^105^ MVMR applies the MR framework to multiple exposures simultaneously, and the method can estimate the direct effect of one exposure conditional on another exposure. *Cis*-MVMR extends the MVMR method to a *cis*-MR setting.^104^ Two-step *cis*-MR uses the product of coefficient method to estimate the extent to which the variant-outcome association is mediated by a potentially pleiotropic (non-*cis*) phenotype and then adjusts MR estimates for effects mediated by the pleiotropic phenotype.^105^

Additionally, some extensions for MR can be applied equally validly in *cis*-MR and genome-wide MR settings. For instance, variant-covariate interactions can be used to assess evidence for pleiotropic effects using the MR-GxE (MR gene-by-environment) framework.^106^ Methods for non-linear MR can be applied both using *cis*- and genome-wide instruments,^107^^,^^108^ and the same cautions apply in both cases.^109^^,^^110^ Factorial MR, which assesses the existence of interactions between treatment-mimicking variants,^111^ is typically implemented with *cis*-instruments.^112^^,^^113^

## Summary

It is tempting to view genome-wide MR analyses, using many genetic variants and sophisticated statistical methods, as reliable and *cis*-MR analyses, potentially using only a single genetic variant, as overly simplistic. However, the opposite is often true: appropriately designed *cis*-MR analyses are generally more likely to fulfill the instrumental variable assumptions and therefore—from a biological perspective—provide more robust evidence of causality than genome-wide MR analyses.^19^^,^^21^ *Cis*-MR analyses using a single genetic variant should not be dismissed—in many cases, using a single variant is the optimal analysis choice.

**Figure 4:**
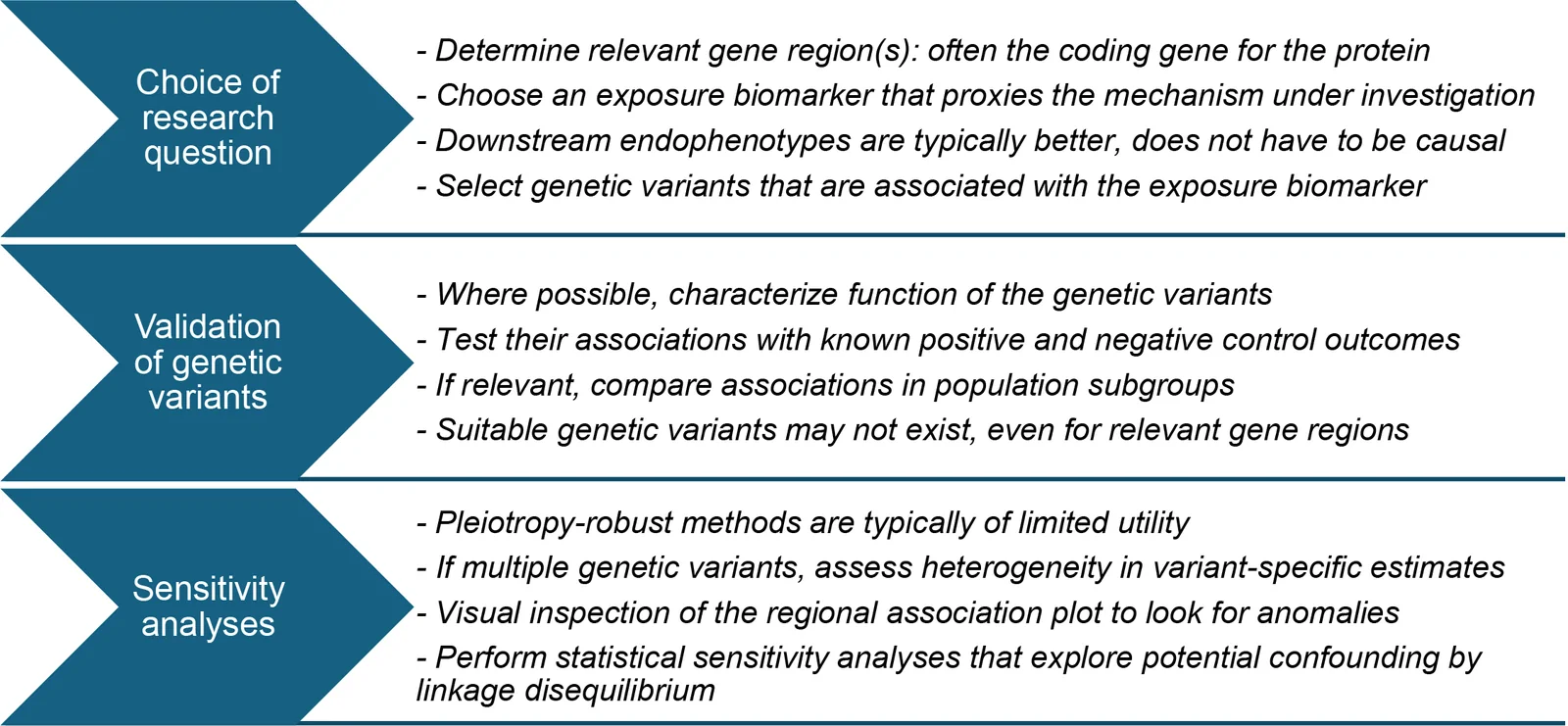
Overview of considerations for *cis*-Mendelian randomization discussed in the paper

Additionally, *cis*-MR involves many methodological considerations when selecting genetic variants, even if the eventual choice is only a single variant. Each *cis*-MR analysis will be different, and the analysis strategy has to be calibrated accordingly; a tailored approach is needed rather than an off-the-peg one. Important decisions involve the choice and specification of the gene region(s) of interest, selection of a valid exposure biomarker to proxy the true exposure, variant selection strategy, validation of the genetic variants, and sensitivity analyses (Figure 4). Although *cis*-MR analyses have often been performed in an algorithmic and high-throughput way for thousands of proteins at a time,^114^^,^^115^ such analyses are unlikely to be optimal and may result in implausible, false positive, or false negative findings depending on whether the selected variant(s) are pleiotropic or irrelevant. Indeed, *cis*-MR studies are not intrinsically superior to genome-wide MR studies, particularly when the selected variants are not adequately assessed as valid instruments.

Any epidemiological analysis requires careful thought, and MR is not an exception; there are no one-size-fits-all solutions for any type of MR study. In *cis*-MR, one must pay specific attention to the biological function of the genomic region of interest and carefully consider the choice of gene region and exposure biomarker, as well as potential sources of pleiotropy. Applying appropriate sensitivity analyses to assess potential violations of the MR assumptions can strengthen the inferences from a *cis*-MR study. Finally, *cis*-MR results should be viewed as one source of evidence that contributes to the answer of a complex question via the triangulation-of-evidence framework^116^ and complements other study designs with different sources of bias.

In conclusion, while *cis*-MR is a powerful tool to inform about putative causal effects, we highlight the importance of incorporating biological insight into *cis*-MR and caution against treating the approach purely as a statistical exercise. We hope that our work will be useful not only to applied scientists conducting *cis*-MR studies but also to peer reviewers and journal editors who assess the quality of those studies during the publication process and that our recommendations will improve the standard of *cis*-MR investigations.

## Data and code availability

The script to reproduce the example analysis is available at https://github.com/vkarhune/cisMRguide.

## Acknowledgments

V.K., B.W., and S.B. are supported by the 10.13039/100010269Wellcome Trust (225790/Z/22/Z) and the UK Research and Innovation Medical Research Council (MC_UU_00040/01). The authors thank Dr. Kaur Alasoo and one anonymous referee for their constructive comments during peer review.

## Declaration of interests

D.G. is the founder and chief executive officer of Sequoia Genetics. Sequoia Genetics is a private limited company that works with investors and pharma and biotech companies, performing research that leverages genetic data to help inform drug discovery and development. V.K. and S.B. are employed by Sequoia Genetics. P.B. is a full-time employee and stockholder at Eli Lilly and Company.

## Supplemental information

Document S1. Figure S1, Tables S1 and S2, and Notes S1 and S2

Document S2. Article plus supplemental information
