# Supplementary material for: Integrating genetic data with biological insight: A practical guide to cis-Mendelian randomization
Source: Am J Hum Genet. 2026 Apr 10;113(5):900–14. doi: 10.1016/j.ajhg.2026.03.011 (PMC13277696; doi:10.1016/j.ajhg.2026.03.011)
Supplement: Document S1. Figure S1, Tables S1 and S2, and Notes S1 and S2 [file mmc1.pdf]

The American Journal of Human Genetics, Volume *113*

Supplemental information

Integrating genetic data with biological insight: A practical guide to *cis*-Mendelian randomization

Ville Karhunen, Benjamin Woolf, Pallav Bhatnagar, Dipender Gill, and Stephen Burgess

Supplemental Note S1

Choosing the exposure biomarker in *cis*-Mendelian randomization

Consider a scenario depicted in Figure S1, where $X_1, \dots, X_k$ may present gene expression in different tissues. The interest is in the causal mechanism (e.g. drug target effect) that is specific to X_1, while the measurements can only be made on another tissue X_2, or on an aggregate level across multiple tissues X_{agg}. Biomarker B is a downstream measure of X_1. Any confounders that affect Xs, B or Y have been omitted for clarity.

Let us indicate β_{AB} as any effect from node A to B. Therefore, the true causal effect of X_1 on Y is $\Gamma = \beta_{X_1Y} + \beta_{X_1B}\beta_{BY}$. If we were to use X_2 to proxy the exposure within the Mendelian randomization (MR) framework, the estimand for the causal effect is

$$\frac{\beta_{GX_1}(\beta_{X_1Y} + \beta_{X_1B}\beta_{BY})}{\beta_{GX_2}} = \frac{\beta_{GX_1}}{\beta_{GX_2}}\Gamma,$$

which is a biased estimand of Γ by a factor $\beta_{GX_1}/\beta_{GX_2}$, which can be positive or negative depending on the signs of β_{GX_1} and β_{GX_2}.

If we instead use the biomarker B as the exposure biomarker, the MR estimand is

$$\frac{\beta_{GX_1}(\beta_{X_1Y} + \beta_{X_1B}\beta_{BY})}{\beta_{GX_1}\beta_{X_1B}} = \frac{1}{\beta_{X_1B}}\Gamma.$$

Therefore, while the causal estimand will be biased by a factor of β_{X_1B}, testing for null hypothesis $\Gamma = 0$ is valid, and the direction of effect will be known as long as the sign of β_{X_1B} is known. Importantly, even if there is no true causal effect of the biomarker B on the outcome Y (ie. $\beta_{BY} = 0$) the estimand remains the same.

Finally, if we use the aggregate measure as the exposure biomarker, the MR estimand is

$$\frac{\beta_{GX_1}(\beta_{X_1Y} + \beta_{X_1B}\beta_{BY})}{\sum_j \beta_{GX_j}}, j = 1, \dots, k,$$

where the direction of bias is untraceable.

Supplemental Note S2

Apolipoprotein C-I signaling and Alzheimer disease

We provide an example of assessing the effect of apolipoprotein C-I (APOC1) signaling on Alzheimer disease risk to showcase the problems of conventional sensitivity analysis in *cis*-Mendelian randomization when there are systematic violations to instrumental variable assumptions. We use total cholesterol levels as the exposure biomarker for APOC1 signaling. Genetic associations with total cholesterol levels were obtained from the genome-wide association study (GWAS) of 1,320,016 European ancestry individuals by Graham et al. (2021)[1]. The GWAS summary statistics were obtained from the Global Lipids Genetics Consortium website: https://csg.sph.umich.edu/willer/public/glgc-lipids2021/results/ancestry_specific/. Genetic associations with Alzheimer disease risk were obtained from a GWAS of clinically diagnosed Alzheimer disease or parental Alzheimer disease diagnosis (71,880 cases, 383,378 controls) by Jansen et al. (2019)[2]. These GWAS summary statistics were obtained from the GWAS Catalog: https://www.ebi.ac.uk/gwas/studies/GCST007320.

To proxy APOC1 signaling, we selected variants associated with total cholesterol levels at $p<5\times10^{-8}$ within *APOC1* (chr19:45,417,504-45,422,606 on genome build 37), clumped at $r^2<0.15$ using 1000 Genomes European ancestry as the linkage disequilibrium reference (downloaded from http://fileserve.mrcieu.ac.uk/ld/1kg.v3.tgz). This procedure resulted in four variants. We harmonized the effect alleles across the exposure and the outcome, and calculated the Mendelian randomization estimates using the inverse-variance weighted, weighted median, and weighted mode methods. The analysis was conducted using R software version 4.3.2. The analysis script is available at: https://github.com/vkarhune/cisMRguide.

Supplemental Figures

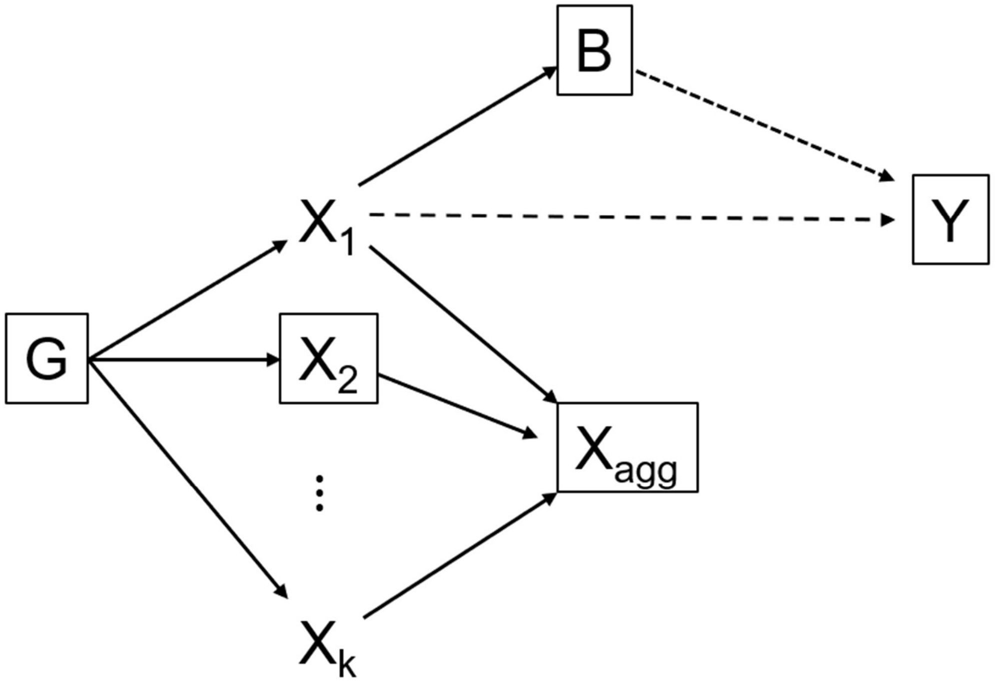

Figure S1: Various options for the exposure biomarker in Mendelian randomization. The causal effect of interest is the mechanism from X1 to Y (dashed line). Boxes indicate measured variables. G refers to genetic variants, and B is the biomarker downstream of the drug target effect of interest. We show that when using B as the exposure biomarker, the null hypothesis for no association is valid, even if the effect of B on Y (dotted line) is zero.

Supplemental Tables

Method	MR estimate (95% confidence interval)
Inverse-variance weighted	3.17 (2.05 to 4.91)
Weighted median	2.96 (2.83 to 3.11)
Weighted mode	2.97 (2.83 to 3.11)

Table S1. Mendelian randomization (MR) results using different methods for the example of apolipoprotein C-I signaling on Alzheimer disease risk.

Variant	r^2 with rs429358
rs12691088	0.12
rs12721046	0.48
rs140480140	0.14
rs484195	0.11

Table S2. Linkage disequilibrium r^2 values (based on 1000 Genomes European ancestry reference) for the variants used to proxy apolipoprotein C-I signaling with Alzheimer disease risk variant rs429358.

Supplemental references

1. Graham, S.E., Clarke, S.L., Wu, K.-H.H., Kanoni, S., Zajac, G.J.M., Ramdas, S., Surakka, I., Ntalla, I., Vedantam, S., Winkler, T.W., et al. (2021). The power of genetic diversity in genome-wide association studies of lipids. Nature *600*, 675–679. https://doi.org/10.1038/s41586-021-04064-3.

2. Jansen, I.E., Savage, J.E., Watanabe, K., Bryois, J., Williams, D.M., Steinberg, S., Sealock, J., Karlsson, I.K., Hägg, S., Athanasiu, L., et al. (2019). Genome-wide meta-analysis identifies new loci and functional pathways influencing Alzheimer's disease risk. Nat. Genet. *51*, 404–413. https://doi.org/10.1038/s41588-018-0311-9.
